# Unobtrusive Vital Sign Monitoring in Automotive Environments—A Review

**DOI:** 10.3390/s18093080

**Published:** 2018-09-13

**Authors:** Steffen Leonhardt, Lennart Leicht, Daniel Teichmann

**Affiliations:** 1 Chair for Medical Information Technology, Helmholtz-Institute for Biomedical Engineering, RWTH Aachen University, D-52076 Aachen, Germany; leicht@hia.rwth-aachen.de; 2 Institute for Medical Engineering and Science (IMES), Massachusetts Institute of Technology (M.I.T.), Boston, MA 02139, USA; Teichmann@hia.rwth-aachen.de

**Keywords:** unobtrusive monitoring techniques, car seat, driver state monitoring, vehicle, electrocardiogram, steering wheel, capacitive electrocardiogram, magnetic impedance, eddy currents, ballistocardiography, photoplethysmography, PPG imaging, infrared thermography, RADAR

## Abstract

This review provides an overview of unobtrusive monitoring techniques that could be used to monitor some of the human vital signs (i.e., heart activity, breathing activity, temperature and potentially oxygen saturation) in a car seat. It will be shown that many techniques actually measure mechanical displacement, either on the body surface and/or inside the body. However, there are also techniques like capacitive electrocardiogram or bioimpedance that reflect electrical activity or passive electrical properties or thermal properties (infrared thermography). In addition, photopleythysmographic methods depend on optical properties (like scattering and absorption) of biological tissues and—mainly—blood. As all unobtrusive sensing modalities are always fragile and at risk of being contaminated by disturbances (like motion, rapidly changing environmental conditions, triboelectricity), the scope of the paper includes a survey on redundant sensor arrangements. Finally, this review also provides an overview of automotive demonstrators for vital sign monitoring.

## 1. Introduction

To assess the state of a patient, clinicians classically employ four primary vital signs [1] for monitoring at the bedside: pulse (heart rate, HR), breathing rate (respiratory rate, RR), blood pressure (BP), and body (core) temperature (BT). Usually, oxygen saturation (SpO_2_) is added as a fifth vital sign, and some authors even propose to add another three vital signs, namely pain, level of consciousness and urine output [2], despite the fact that they are more difficult to measure continuously.

These classical methods to measure vital signs typically require close physical interaction with the patient (cables, electrodes, cuffs and pneumatic lines, etc.), making the measurement suitable for a clinical environment, but limiting its usability for applications outside the hospital. Therefore, technologies for unobtrusive and contact-free monitoring of vital signs have attracted a lot of attention in recent years [3,4,5]. In fact, such techniques would open up completely new applications, including home care and sports, but also supervision during operation of vehicles.

Due to its impact on road safety and casualty statistics, car driver state monitoring has been an active area of research for a long time, and physiological sensors like electrocardiogram (ECG) and electroencephalogram (EEG) have often been employed in trials. For many years, researchers looked at e.g., stress [6,7] and drowsiness [8]. Based on video cameras and classical image processing technologies instead of physiological sensors, however, especially drowsiness and fatigue detection have been targeted much longer and relied on, for example, eye blink analysis and percentage of eye closure (PERCLOS) assessment (see [9,10]).

From a practical perspective, cable-bound solutions are not an option for driver state monitoring outside experimental trials. Hence, unobtrusive and non-contact driver state monitoring using physiological information is currently gaining increasing attention [11]. This trend is pushed in part by the ongoing R&D efforts towards autonomous driving and also by the demographic changes producing more elderly and potentially diseased drivers [12]. However, while simple schemes like the analysis of steering patterns to assess driver state have already been commercialized by several car manufacturers, the analysis of true physiological information coming from ECG, EEG and photoplethysmography (PPG) sensors still is in the research stage [13].

Hence, to support the ongoing developments of the scientific and industrial community, this review paper summarizes the state of the art in vital sign monitoring for this challenging application and discusses maturity as well as pros and cons of the individual concepts. It also points at potential solutions to increase coverage and vital sign accuracy resulting from motion artifacts, namely artifact compensation and/or signal and sensor fusion.

## 2. Unobtrusive Sensing of Physiological Parameters and Features

As mentioned above, the term *vital signs* typically refers to five physiological parameters: HR, RR, BP, BT and SpO_2_. Since no attempts have been conducted to equip cars with unobtrusive BP monitoring, this vital sign will not be further addressed in this review.

In 2012, Scalise et al. [14] presented a comprehensive overview of contact-based (ECG and PPG) and non-contact (CW and PW (UWB) radar, laser vibrocardiography [15], magnetic induction, capacitive ECG, ultrasound transmitter) heart monitoring systems.

In 2014, Kranjec et al. [16] presented a review on heart rate and heart rate variability measurements. Apart from conventional measurement approaches (ECG and PPG), experimental approaches were also presented, including the measurement by thermal imaging, imaging using visible light, optical vibrocardiography, doppler radar, capacitive ECG and heart rate extraction from speech. Advantages and disadvantages of the experimental approaches were discussed. In the same year, Kranjec et al. [17] presented a feasability study for several experimental heart rate measurement methods. The authors separated the methods into two categories, depicted as “direct methods” measuring the electrical heart activity (capacitive ECG) and “indirect methods” measuring mechanical displacement of the patients body (microwave and ultrasound distance measurement, signal from microphone).

**Figure 1:**
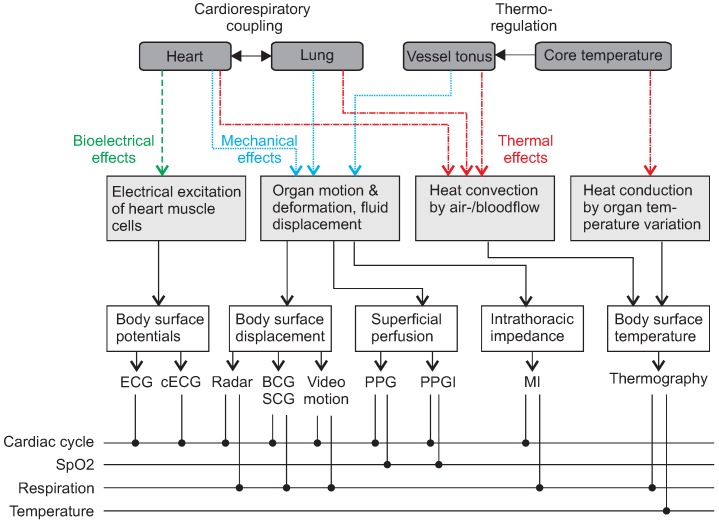
Overview of physiological sources, effects, unobtrusive and non-contact sensors, and obtainable vital signals, modified and extended from [3] (see Figure 1).

In 2015, Brüser et al. [3] from RWTH Aachen University provided a methodological review on nine different non-contact sensing modalities targeting the monitoring of cardiorespiratory activity. As described in this paper, there are three main categories of physical effects that can be sensed unobtrusively: bioelectrical, mechanical and thermal effects. Figure 1 provides an overview of the different effect categories, and how they relate to organ activity as well as to the measured vital signs.

The activity of the heart can be observed by measuring body surface potentials which are generated by the electrical excitation of heart muscle cells. The measurement of these body surface potentials is called ECG or in the case of capacitively coupled measurement capacitive ECG (cECG), see Section 3, and provides extensive information about the cardiac cycle.

Any intrathoracic organ motion will have mechanical effects. Such mechanical effects include the displacement of organ boundaries, deformation of surfaces and the displacement of fluids and may modulate the displacement of the body surface and the superficial perfusion, but also the intrathoracic impedance distribution. Body surface displacement can be obtained by radar, ballistocardiography (BCG), seismocardiography (SCG) (see Section 4) or video (see Section 5) and has been used for HR as well as RR monitoring. The superficial perfusion and its effect on the optical properties of the skin is the basis for PPG and PPG imaging (PPGi). These techniques provide information about the cardiac cycle and SpO_2_ and are described in detail in Section 5. While variations in intrathoracic impedance distribution can also be observed with bioimpedance spectroscopy (BIS) and impedance cardiography (ICG), up to today only magnetic induction (MI) measurements allow unobtrusive (i.e., electrode-free) impedance analysis. In fact, MI has already shown its capability to measure HR and RR (see Section 6).

The body surface temperature, which can be monitored in an unobtrusive way by radiation-based thermography as described in Section 5, is modulated by changes in the body’s core temperature by direct conduction of heat between the organs and blood to the skin. Next to this, the body surface temperature is also modulated by heart and lung activity via heat convection by blood and air and, therefore, allows RR monitoring. In principle, body surface-based HR measurement could also be possible, but today’s thermography devices do not provide the necessary sensitivity.

Figure 1 covers unobtrusive sensors and the underlying physiological effects in general. Correspondingly, in the following chapters, we will cover the historical developments and the state-of-the art of these technologies in more detail, but specifically illuminate the current state of unobtrusive sensing inside a car.

## 3. ECG Monitoring in a Car

The electrocardiogram (ECG) is a biopotential, with the body floating with respect to the ground. Hence, its measurement requires at least two electrodes and a differential amplifier. The ECG reflects electrical and—to some extent—mechanical function of the heart muscle. It is fair to say that it is the most valuable physiological signal when it comes to heart diagnostics [18,19].

Classical ECG monitoring employs well-conducting adhesive electrodes made from silver with a thin silverchloride surface coating being in direct contact to the skin. Such electrodes are coupled to the body surface by well-conducting gels and are typically cable-bound, which prohibits regular use in non-clinical scenarios. Electrochemically, such electrodes are termed *non-polarizable*. The combination of a difficult to dissolve metal and its metal chloride make these electrodes less sensitive to ion concentration changes and hence their polarization is very stable. The electrode mainly behaves like a resistor.

Plain metal electrodes (like stainless steel) are different in that they represent *polarizable* dry electrodes. While cheap and simple, their polarization depends on the salt concentrations of the surroundings (e.g., skin) and hence their polarization is not stable. Electrically, such electrodes mainly behave like capacitors.

Low conduction or capacitive electrodes have no direct galvanic contact to the body. Typically, capacitive electrodes are separated from the body by cloth or other textile layers. As the measured ECG signals are at least one order of magnitude smaller than with galvanic electrodes, capacitive electrodes typically have an operational amplifier directly at the electrode. The compensation of common mode by an active driven ground is much more important than in the galvanic case.

**Figure 2:**
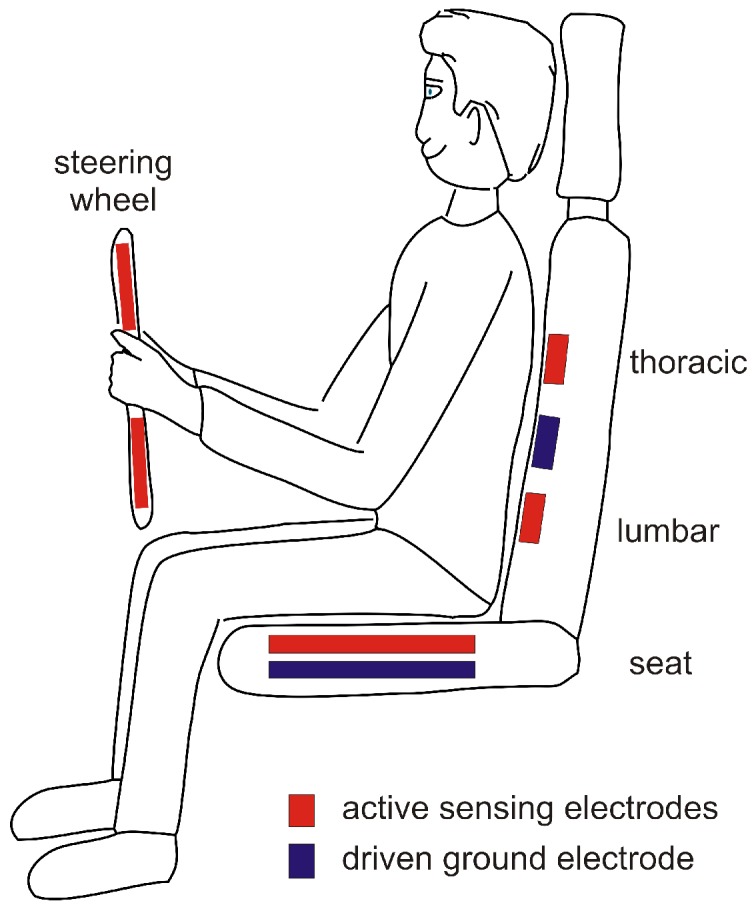
Electrode locations for conductive and low-contact ECG monitoring around a car seat. Red blocks indicate tested locations for sensing electrodes, blue blocks indicate published locations for the driven ground electrode.

For the car, electrodes can be placed at various locations, including the steering wheel (conductive), the backrest (capacitive, both thoracic or lumbar) and the seat (capacitive). For reduction of common mode, driven ground electrodes can be added. While thoracic placement of low-contact electrodes has the advantage of a larger signal strength, this location may also be the one which is subject to the biggest motion artifacts. Figure 2 gives an overview of published electrode locations for heart rate monitoring in the car.

### 3.1. Conductive ECG Monitoring

Placing electrodes on the steering wheel has been one of the early research targets. The available literature dates back at least a decade. In an attempt to quantify driver stress levels, in 2007, Jeong and colleagues [20] from Yonsei University in Korea investigated driver task load and stress response looking at heart rate variability (HRV) obtained with a steering wheel-based pair of dry ECG electrodes.

Similarly, in the same year, Lee and coworkers from Seoul National University in Korea reported about their study using dry electrodes integrated into the steering wheel [21]. These tape-type dry electrodes were made of copper tape (3M). Three male students were recruited as test drivers and asked to drive a route of 16 km in about 40–50 min. Heart rate variability was assessed using a modified Tompkins algorithm for R-peak detection and computation of R-peak intervals, heart rate, SDNN, RMSSD and pNN50. While just an abstract without providing any details, Hu and colleagues also reported on the integration of ECG into the steering wheel in 2008 [22].

In 2010, Shin et al. [23] and later Jung et al. [24] from Pukyong National University in Busan, Korea, presented steering wheel electrodes based on conductive fabric. In parallel, a multisensor system allowing to measure SpO_2_, temperature and skin temperature at the steering wheel of a Mercedes S-Class vehicle (DAIMLER AG, Mercedesstraße 137, D-70327 Stuttgart, Germany) was published by Heuer and colleagues [25] from Karlsruhe Institute of Technology, Germany. Around the same time, D’Angelo and colleagues from the Technical University of Munich demonstrated a multisensor system integrated into the steering wheel of a BMW 730d test vehicle (BMW AG, Petuelring 130, D-80788 München, Germany) [26,27] featuring SpO_2_ and conductive ECG sensing as well. The authors used a design similar to [20], integrating the electrodes into the steering wheel, the shift lever and the left arm rest. However, the precise electrode position was not revealed in this work.

In 2012, Gomez-Clapers and Casanella [28] from the Universitat Politecnica de Catalunya in Barcelona, Spain, presented a steering wheel-based ECG-measurement demonstrator which was based on dry ECG electrodes and wireless communication. Similarily, in that year, Silva and colleagues [29] from IT/IST-UTL in Lisbon, Portugal, demonstrated that dry Ag/AgCl electrodes on a steering wheel can produce ECG measurements of similar quality as traditional electrodes.

Another Korean project from Pukyong National University was presented in 2014. Jung and colleagues [24] proposed a design featuring a steering wheel with two conductive fabric electrodes, similiar to [29]. This electronic circuit design was based on an MSP 430 microcontroller and a Chipcon CC2420 RF Transceiver (both manufactured by Texas Instruments Inc., P.O. Box 660199, Dallas, TX, USA) for wireless data transmission at 2.4 GHz.

In 2015, Eßers et al. of Takata corporation (Takata corporation, Tokio, Japan) presented a steering wheel with sensors measuring the heart frequency. The actual integration is not well documented in the publication; it can only be assumed that it is similar to Lee [21] and Silva [29].

**Figure 3:**
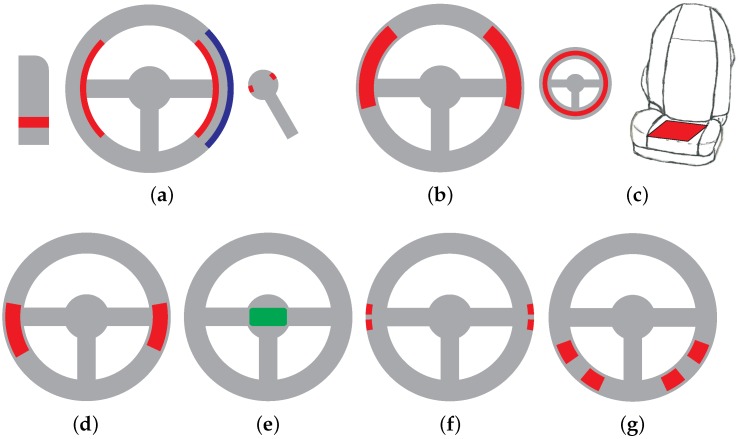
Overview of several designs for contact-based ECG monitoring (red: active electrodes, blue: driven ground electrode, green: electronics, precise electrode position on steering wheel not revealed): (**a**) Jeong [20]; (**b**) Lee [21], Silva [29], Vavrinsky [30,31]; (**c**) Matsuda [32], Xu [33]; (**d**) Heuer [25]; (**e**) D’Angelo [27]; (**f**) Gomez-Clapers [28]; (**g**) Jung [24].

Figure 3 gives an overview of different electrode placements.

We note that ECG monitoring using steering wheel-based approaches is a feasible option for heart rate tracking but requires both hands to touch two different conductive parts of the wheel. Hence, significant steering movements with changes of the location of gripping and the quite common habit of steering with one hand pose a problem for this technique.

#### Hybrid ECG Monitoring

A somewhat different research approach was followed by Matsuda and Makikawa from Ritsumeikan University in Shiga, Japan. In 2008, these authors proposed using a conductive steering wheel and combine it with a capacitive electrode located in the driver seat [32] (see Figure 3c). This way, the habit of steering with one hand can be better accomodated. This proposal also combines the concepts of conductive measurements with capacitive sensing, which may justify the name *hybrid sensing*.

One year later, the idea of *hybrid ECG sensing* using the steering wheel as one electrode and the seat as the second (capacitive) electrode, was also proposed by Baek and coworkers, see [34], next to redundant sensing and sensor fusion.

In 2013, the idea of a steering wheel electrode measuring against a capacitve electrode in the seating area was again presented by Xu and Ta [33] from the Central South University, Hunan, China, possibly without knowledge of Matsuda’s work.

### 3.2. Capacitive ECG Monitoring

Unlike the classical conductive electrodes, low-contact electrodes do not rely on a galvanic skin contact, but achieve the signal coupling by means of a high-impedance or even capacitive coupling. In the case of capacitive electrodes, the surface is electrically insulated and remains stable in long-term applications. This concept is of course only of theoretical nature, since a purely capacitive electrode would require an infinitively high ohmic resistance—which no real electrode can provide. Thus, all existing capacitive electrodes are in fact low-contact electrodes. Consequently, they should also be named as such. However, in most of the recent literature, they are entitled as capacitive electrodes. For the sake of consistance, we will also use the term capacitive electrode in that synonymous sense, describing an electrode with a high-impedance coupling to the patient.

A timeline of the development of capacitive ECG systems is given in Table 1. Capacitive (insulated) electrodes date back more than 50 years and were first described by Richardson [35]. Early work regarding the integration of capacitive ECG measuring systems into objects of daily life (beds) was performed by Ishijima [36]. In his work, the author used conductive dry textile electrodes acting as an underlay or pillow. Proper ECG detection during sleep was reported. However, due to potential direct skin contact, the patient’s coupling may not have been exclusively capacitive.

After the millenium, Park and coworkers from Seoul National University in Korea began to explore integration into other objects of daily life. For example, in 2004 Lim et al. demonstrated the integration of capacitive electrodes into a bathtub [37] and also in 2004 a toilet seat was presented [38]. In 2006, this group published the integration of capacitive ECG electrodes into a chair [39]. Later, Lim et al. [40] and also Wu and Zhang [41] reported about integration of dry and capacitive ECG into beds.

At RWTH Aachen University in Aachen, Germany, the first applications of capacitive ECG electrodes were published in 2006 [56] and 2007 [57]. The so-called *Aachen Smart chair* is an office chair equipped with two solid copper plate electrodes integrated into the backrest. Beneath the seating area, hidden by the commercial textile cover, a large driven ground electrode is located. It acts as an active reference electrode. The backrest electrodes are coated by a protective black acrylic paint to achieve a purely capacitive coupling. Note that a similar design has been proposed in 2006 by Lim et al. [39].

In June 2008, capactive ECG monitoring reached the automotive domain, when the first paper proposing capacitive ECG sensing while driving a car was published by Leonhardt and Aleksandrowicz [42]. In this particular design, solid copper plate electrodes coated with a protective acrylic paint were placed on the backrest of a car seat at a 45° angle, with the aim of improving the signal-to-noise ratio (SNR) by simulating a typical heart axis. A *driven right leg* electrode (referred to as *driven ground plane*) was also placed in the backrest around the electrodes, as in this design the seat was already carrying an EMFi^TM^ foil (Emfit Ltd., Konttisentie 8, FI-40800 Vaajakoski, Finland) for simultaneous BCG measurements.

**Figure 4:**
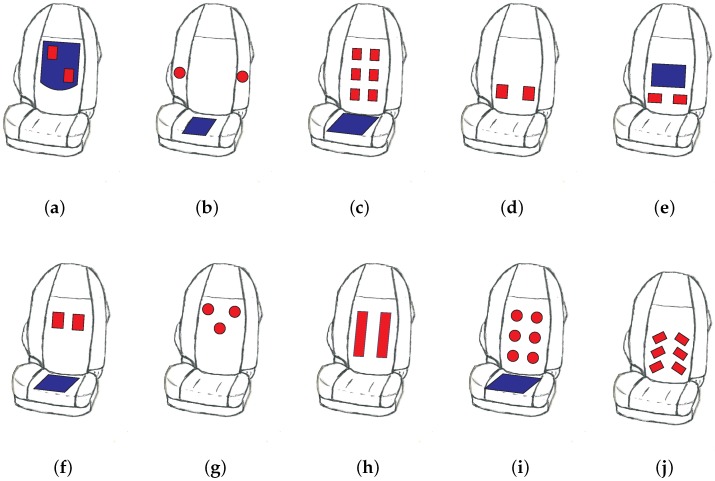
Overview of several low-contact ECG electrode arrangements and their year of introduction to the public (red: active electrodes, blue: driven ground electrode). (**a**) SMART seat (Leonhardt, 2008) [42]; (**b**) Daimler S-Class (Chamadiya, 2008) [43]; (**c**) Ford S-Max (Eilebrecht, 2011) [44]; (**d**) Daimler car (Chamadiya, 2011) [58]; (**e**) Audi Q5 (Schneider, 2012) [48]; (**f**) Car seat (Jung, 2012) [49]; (**g**) EPIC System (Plessey, 2014) [59]; (**h**) Ford S-Max (Leicht, 2014) [60]; (**i**) Ford car (Leicht, 2015) [61]; (**j**) WARDEN (Plessey, 2017) [52].

This design can be seen as one of the ancestors from which, over the years, a large variety of seats have evolved. Apart from differences in the the design of the electronics, they differ by the number of electrodes, their size, shape, location and material. Most, but not all, designs employ a driven reference electrode. An overview of several designs and their release date is presented in Figure 4.

Later in 2008, Chamadiya and colleagues from the Karlsruhe Institute of Technology (KIT) presented a concept integrating textile capacitive electrodes into a W221 Mercedes S-Class seat [43]. In this specific design, the electrodes were placed horizontally and rather lateral as the chosen seat already carried several other technical devices (ventilator, massage unit). However, this lateral electrode placement seems suboptimal as it may result in reduced body contact areas and subsequent lower coverage.

In 2011, Eilebrecht and colleagues from RWTH Aachen University published a multi-electrode design with six rectangular metal plate electrodes integrated into the backrest of a Ford S-Max driver seat [44]. This arrangement has the advantage that, when looking at different electrode pairs, selection of the strongest signal is possible. Implicitly, this allows for adapting to different heart axes and also to the torso size of different drivers.

With *n* = 7 test drivers, the practical usability of this design was tested on the Ford proving ground in Lommel (Belgium). Overall, ECG coverage during driving was 85% with three layers of clothing and moved up to 93% when limiting the allowed clothing to two layers [44]. Urban driving was found to be more challenging than smooth driving on a German highway. In specific drivers, the nervous reaction to fear (increased heart rate) and dedicated pathologies like extrasystoles were clearly visible.

Using this seat, Wartzek et al. compared the ECG measurement performance and coverage during Highway and city traffic in *n* = 5 test drivers [45]. Later in that year, the same group [46] described in detail that motion artifacts and especially their effective combination with local as well as global triboelectricity play an important role in capacitive monitoring and to date limit the robustness of this technique.

In a joint effort by the Karlsruhe Institute of Technology (KIT) and Daimler (DAIMLER AG, Mercedesstraße 137, D-70327 Stuttgart, Germany), textile integration into the car seat was further demonstrated by Chamadiya et al. in the same year [58]. A two electrode design featuring two textile electrodes placed horizontally and rather lumbar has been presented which provided decent ECG measurements in pilot trials.

Around that time, Schumm and colleagues from ETH Zurich in Switzerland obtained cECG test equipment from the Philips Chair at RWTH Aachen University. Based on the European SEAT project (EU 6th Framework Program, contract number: AST5-CT-2006-030958), the authors investigated if capacitive ECG measurements would be feasible for passenger monitoring in an airplane seat [47,62]. In their 2012 publication, Schumm et al. pointed out that the signal quality of unobtrusive sensing during various passenger activities might not always be sufficient for continuous monitoring [47]. The authors hence introduced a signal class label that reflects the signal quality and called it the *quality label* (QL).

In addition in 2012, Schneider et al. from the FZI Research Center for Information Technology in Karlsruhe, Germany, developed a framework for vital sign measurements inside a vehicle including a textile seat cover [48]. The design features two textile electrodes located horizontally in the lumbar region of the back and a driven seat electrode above the two active sensing electrodes. The design was demonstrated in an Audi Q5.

In the same year, Jung and coworkers from Pukyong National University presented another capacitive ECG monitoring system with two horizontal electrodes integrated into a car [49]. In this design, the active measurement electrodes were again planar copper plates located horizontally in the backrest, while the driven right leg electrode was made of conductive fabrics and located on the seat. In the presented trial, the authors investigated HRV analysis on measurements obtained from *n* = 5 subjects without any heart disease.

At the 2012 EXPO in Chicago, IL, USA, Plessey Ltd. (Plessey Semiconductors Ltd., Plymouth PL6 7BQ, UK) announced the availability of their capacitive ECG sensor system EPIC*™*. About two years later, the EPIC*™* system was tested in an office chair [63]. In addition, a three-electrode system integrated into the car seat was announced in the media [59].

In 2014, Leicht et al. demonstrated that a local release of water vapor can dramatically increase the SNR of capacitive ECG [60]. As an underlying principle, it was shown that the increase of local air humidity can improve the ohmic coupling (hence the coupling will not be exclusively capacitive anymore). At the same time, this allows static charge difference induced by triboelectric effects to flow to ground. It hence reduces the impact of triboelectricity on capacitive monitoring. Leicht et al. proposed using conductive textile electrodes that are permeable for water vapor and to place chambers with silica gel as a source or sink for humidity behind the textile electrodes, but also other concepts for humidification are conceivable [64]. Figure 2h presents the arrangement of the electrodes used for prooving the concept.

In 2015, the RWTH Aachen group presented a multi-electrode design integrated into a Ford S-Max commercial test vehicle (Ford Motor Company, Dearborn, MI, USA). While in this design the electrodes remained solid metal, their shape was changed to round and the contract area was reduced as compared to previous designs. Note that the textile driven ground has now been completely integrated into the seat. In the same year, some of these authors started clinical trials with survivors of heart attacks or heart surgery in the Rosenquelle rehabilitation hospital in Aachen. Results from this study have been published in [55,61]. In this trial, patients (*n* = 10) were asked to drive in a driving simulator equipped with capacitive ECG in the seat and classical ECG monitoring for reference.

In addition in 2015, the approach of electrode humidification was evaluated by other groups. Fong and Chung [50] described special electrode designs allowing to release some humidity directly at the spot. Weder et al. [51] presented a textile breast belt incorporating a water reservoir used to moisten the electrodes. However, while highly integrated, these concepts are open-loop and provide no definition of the acting local humidity.

Hence, in 2017, Leicht et al. from RWTH Aachen University disclosed a closed-loop concept for feedback control of local humidity [53]. As storage and release of water vapor in silica gel is dependent on the local water vapour pressure, but is also a function of temperature, we proposed to establish closed-loop temperature control as a method to release water vapor from the gel and hence to enhance SNR. The corresponding car seat demonstrator is shown in Figure 4h.

In 2016, Plessey introduced WARDEN^TM^, a six-electrode seat cover including a driven ground electrode built into the main electronic unit located at the opposite site of the backrest [52]. It can be used to easily retrofit car seats for contactless ECG recording and connects to in-vehicle data systems via Bluetooth. In late 2017, a six-electrode design featuring round capacitive electrodes in the backrest was presented to the public by the Belgian research organisation IMEC [54,65]. The overall design has a similiar look as the design presented in [60]. However, in this specific seat, radar sensors were integrated as well, which allows sensor fusion (see Section 9).

To simplify any comparison of designs, Table 2 compares specific features of capacitive ECG electrode placements for car seats as reported in the literature to date.

## 4. Ballistocardiography

Ballistocardiography (BCG) is an old medical sensing technique, with early BCG recordings dating back more than 100 years [66,67]. BCG allows monitoring of cardiac (and usually respiratory) activity unobtrusively. Typically relying on external pressure or strain-gauge sensors, a ballistocardiograph records the vibrations which are caused by the mechanical activity of heart and lung, as well as the momentum of the blood pulse traveling down the aorta. Hence, the diagnostic information is completely different from a classical electrocardiogram (ECG) which records electrical activity of the heart muscle.

In the late 1950s, Scarborough et al. introduced different categories of BCG measurements [68] and defined three axes of measurement for translational BCG recordings: longitudinal (head-foot, also called *craniocaudal*), *transversal* (side to side), and *dorsoventral* (back to chest). We note that typically the craniocaudal momentum is much bigger than the transversal and the dorsovental component. We also note that, to measure a BCG signal, contact (mechanical coupling) is required.

**Figure 5:**
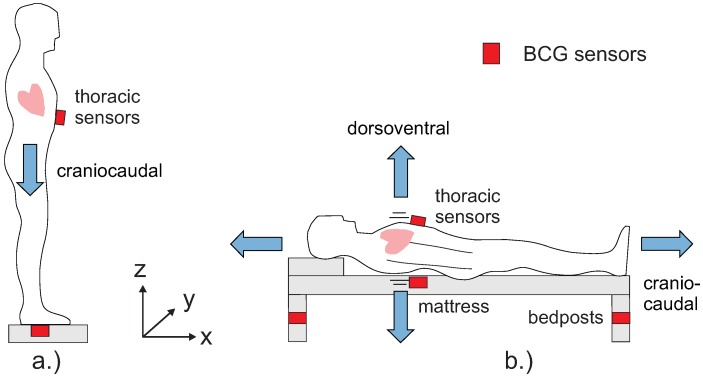
Potential BCG sensor locations: (**a**) measuring cardiac acitivity on a weighing scale (craniocaudal component of the BCG momentum); (**b**) measuring both cardiac and respiratory activiy in bed (dorsoventral component of the BCG momentum).

The early BCG recording systems were typically of the longitudinal kind [66,67]. For a standing person, this corresponds to the *z*-axis (see Figure 5a). Weighing scales which recently received increased attention [69] also measure along this axis. However, bed-based BCG systems [70] measure along a combination of the transversal and dorsoventral axes (see Figure 5b). If the coordinate system is not rotated with the person, but considered a fixed base coordinate system, then the dorsoventral axis corresponds to the *z*-axis as well.

For the bed, sensors can be placed above or between mattresses (for example, quasi-piezoelectric ferroelectret foils), inside the mattress (optical sensors), on the slatted frame (employing e.g., strain-gauge sensors) or in the bedposts (using, for example, pressure sensors).

If pressure or accelaration sensors are attached to the chest to record the heart motion (“thoracic sensors” in Figure 5), the resulting special form of BCG signal is referred to either as

- Phonocardiogram, or
- Apexcardiogram, or
- Kinetocardiogram, or
- Seismocardiogram.

BCG has gained renewed interest in recent years [71], mainly because of the simple and cheap instrumentation and the potential of this technique being used outside classical medical scenarios, for example in sleep analysis. In 2015, a review on recent advances was provided by Inan et al. [72].

However, the BCG signal is much more variable than the ECG signal and also dependent on the position of a (sleeping) body. Due to this high inter- and intravariability, proper heart rate detection is more difficult than ECG analysis. Despite these problems, a very powerful algorithm for individual heart rate detection was proposed by Brüser et al. [73].

**Figure 6:**
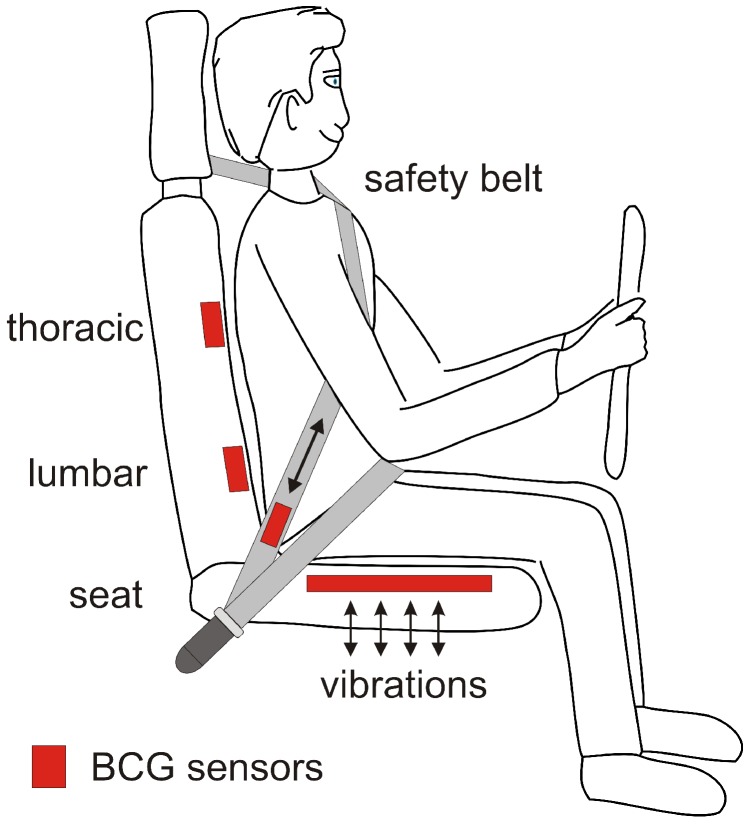
Potential BCG sensor locations in a car seat. Note that the lumbar and especially the thoracic sensor location in the backrest are likely to have contact problems. By contrast, sensors in the seating area as well as in the safety belts will face vibrations coupled from the vehicle body.

The application of BCG sensing for driver monitoring has a lot of similarity with BCG monitoring in a chair. Generally, in this position, the craniocaudal BCG signal component is measured, while the dorsoventral signal component is much more difficult to measure as there may not always a proper mechanical coupling between the torso and the backrest, especially for thoracic sensor locations. Figure 6 sketches the options.

Early attempts to integrate BCG sensors into car seats started about 10 years ago. In 2008 and again in 2011, the placement of BCG sensors in the seating area of a SMART*™* co-driver seat (DAIMLER AG, Mercedesstraße 137, D-70327 Stuttgart, Germany) was successfully demonstrated [42,74]. In this demonstrator, a quasi-piezoelectric ferroelectret EMFi^TM^ mat (Emfit Ltd., Konttisentie 8, FI-40800 Vaajakoski, Finland) was employed as a BCG sensor. However, data supplied in [74] indicates that motor vibrations may make BCG monitoring in a running car difficult.

In order to fight driver fatigue (which is said to account for 20–35% of serious accidents), the HARKEN (HARKEN: Heart and respiration in-car embedded non intrusive sensors, funded by the European Union Seventh Framework Programme (FP7/2007-2013) under grant agreement no.: 286265) project proposed to monitor both cardiac and respiratory activity by means of smart materials embedded in the seat cover and the safety belt of the car [75]. However, no further results on these BCG principles were published.

In 2015, the Faurecia Group (Faurecia S.A., Nanterre, France), a large automotive supplier, presented a car seat called *Active Wellness*^TM^ [76], which claims to detect the traveler’s drowsiness or stress level and then takes countermeasures to relieve the driver from these conditions [77]. The underlying sensing concept employs strain-gauge BCG sensors in the seating to detect cardiac and respiratory activity of drivers and/or occupants.

Very recently, in 2018, Wusk and Gabler presented heart and respiratory rate estimation obtained with a BCG sensor in a Ford Mustang passenger seat [78]. As a sensor, the authors proposed using a “fluid filled bladder connected to a solid-state pressure transducer”. In their study, data from eleven volunteers were collected. Under these controlled laboratory conditions, the authors were able to extract both vital signs with decent accuracy.

## 5. Optical Methods

Radiation has the advantage of unnoticeable transport of information from a driver or passenger without contact. Hence, as long as the optical pathways are free, optical modalities potentially offer unobtrusive sensing from a distance that is attractive for automotive applications, especially since, in modern cars, cameras are already used for external obstacle detection, etc.

**Figure 7:**
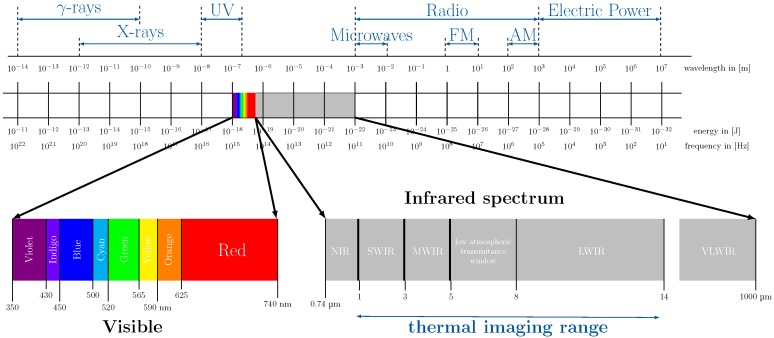
Frequency ranges (VIS, NIR, FIR) usable for optical monitoring techniques.

In the recent past, there has been a boom in camera-based-technologies and applications for vital-sign monitoring. We note that, according to the utilized frequency ranges, the individual camera technologies differ with respect to materials, prices and size. Figure 7 highlights the frequency spectra suitable for cameras.

From Figure 7, we can conclude that there are three frequency ranges suitable for driver status monitoring: visible light (VIS, wavelength 350–740 nm), near-infrared light (NIR, wavelength 740–1000 nm) and far-infrared light (FIR, wavelength > 1 μm). Table 3 compares specific features of the different camera concepts and designs reported in the literature to date.

In the VIS and the NIR range, light can interact with living tissues. Due to conservation of energy, there are three principal modes of interaction: absorption, reflection and transmission. Transmitted light may travel through the tissue on a straight pathway (as in transmissive PPG), but may also be scattered. In fact, in skin tissue, the dominating effect of tissue–photon interaction is scattering (about 50 times stronger than absorption).

**Figure 8:**
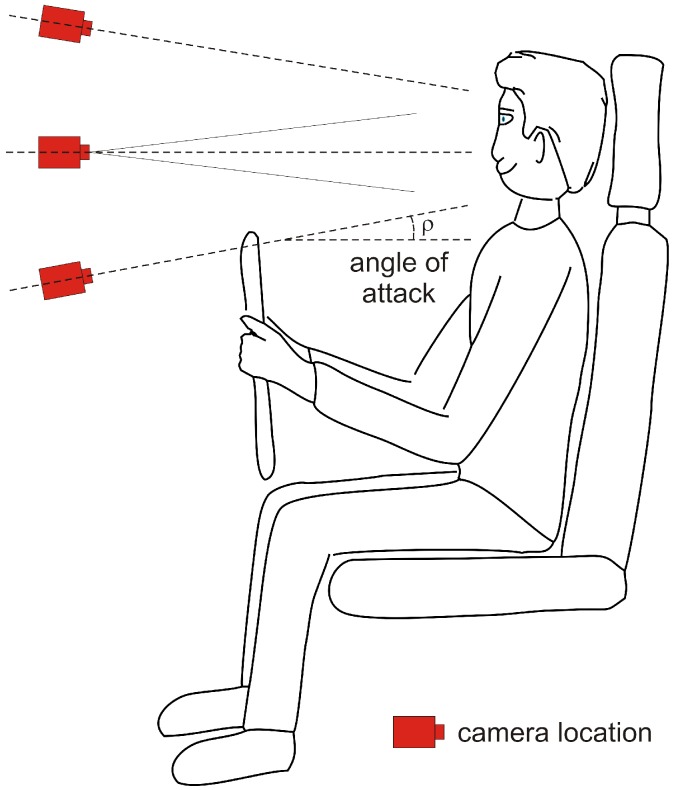
Proper locations for cameras. Locations can be differentiated by the angle of attack ρ.

As for any optical method, a camera-based driver or passenger monitoring inside a car requires free line of sight. As the face offers uncovered skin, looking at the face of drivers and passengers easily comes to mind. Since current cockpit design requires free line of sight for the driver, in addition, the following camera locations have been explored (see Figure 8).

If the angle of attack ρ > 0, the nostrils may become visible, which is interesting for FIR respiratory monitoring. We note that FIR imaging is often referred to as *infrared thermography* (IRT).

### 5.1. Photoplethysmography

**Figure 9:**
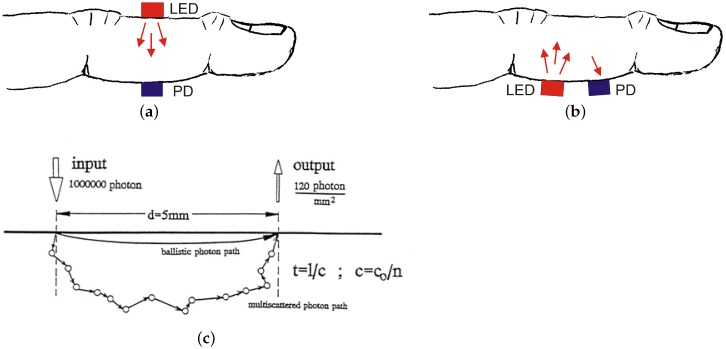
LED and PD placement for transmissive and reflective photoplethysmography. In reflective mode, on average only 50 out of $10^{6}$ photons leave the tissue to reach the PD. (**a**) transmissive PPG (tPPG); (**b**) reflective PPG (rPPG); (**c**) typical banana-shaped pathways of scattered photons in rPPG.

Photoplethysmography (PPG) is a well-known technique allowing measurement of blood volume changes in the skin or in a small part of the body [79]. In classical (local) PPG, the light source typically is a light-emitting diode (LED, either in the VIS and/or NIR frequency range) while the sensor is a photodetector (PD). In *transmissive* PPG (tPPG), the light transmitted through the tissue is detected by a photodetector opposite the light source, while in *reflective* PPG (rPPG), the photodetector detects light that is scattered and/or reflected from the skin (see Figure 9).

Note that, for tPPG, transmission length is limited to a few mm up to cm (allowing typical locations like earlobe or finger), while, in rPPG, penetration depth typically is up to 3 or 4 mm. Reflective PPG relies on sufficient ambient or actively injected light which is then modulated by blood flow in the upper and lower plexus of the skin capillaries. As a result, on their (typically banana-shaped) way through the skin, about 1 out of 20,000 photons reaching the skin from the light source will finally follow a path that allows them to exit from the skin and fly back to the detector collecting this flow of photons.

#### PPG Monitoring in the Car

In 2010, the integration of reflective PPG monitoring into the steering wheel of a BMW 650i test car was demonstrated by d’Angelo and colleagues [26]. The authors used a reflective pulse oximetry sensor connected to an OEM III module manufactured by Nonin Medical (Nonin Medical Inc., 13700 1st Avenue North, Plymouth, MI, 55441-5443 USA). In the same year, Heuer et al. demonstrated SpO_2_ sensing in the steering wheel of a Mercedes S-Class demonstrator cockpit (DAIMLER AG, Mercedesstraße 137, D-70327 Stuttgart, Germany) [25], next to ECG monitoring in the steering wheel and capacitive ECG monitoring in the seat. These authors employed a Nonin 8000R reflective probe. Last but not least, also in 2010, Shin and colleagues [23] reported on a steering wheel design featuring conductive ECG fabrics and reflective PPG using a single frequency (infrared light at 940 nm).

However, a general drawback of this approach is that 100% sensor coverage can not be guaranteed. When integrating reflective PPG sensors into the wheel, the driver has to voluntarily touch/cover the hidden PPG sensor with perfused skin. Hence, this technique may be especially useful for on-demand applications.

### 5.2. PPG Imaging

**Figure 10:**
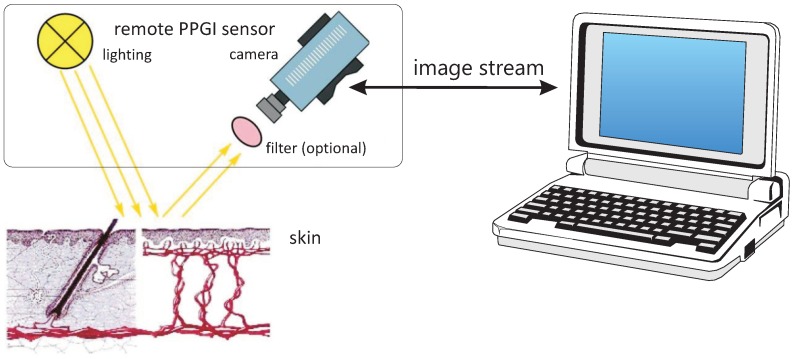
Principle of PPG imaging (PPGI).

Using a camera and a proper light source (VIS and/or NIR, either ambient or artificial illumination), the reflective PPG principle can be expanded to remote camera-based monitoring from a distance. Figure 10 explains the principle.

Note that both the light source and the camera can now operate remotely from a distance but require a free line-of-sight.

Camera-based PPG methods have recently attracted increasing scientific attention. The principle of PPG imaging is a simple one: as the heart beats, the apparent colour of the skin changes due to blood pulsation. In principle, remote PPG imaging allows unobtrusive measurements of heart rate and derived parameters like heart rate variability (HRV) or pulse transit time (PTT) [80], for example when different locations are related to each other or when combined with ECG.

To our knowledge, the earliest PPGI applications were published by Blazek and colleagues from RWTH Aachen University starting in 2000 and centered around heart rate and perfusion measurements at the leg [81,82,83].

In 2005, Wieringa et al. [84] reported on first steps towards SpO_2_ imaging using a multi-wavelength approach. Employing a time-lapse image acquired from a CCD camera, Takano and Ohta [85] developed a non-contact and non-invasive device in 2007, which was able to measure both the respiratory and pulse rate simultaneously. In the same year, Zheng and Hu reported [86] that by using LEDs at three wavelengths (VIS and NIR), they were able to extract a PPG signal from skin with a CCD USB camera.

In 2008, Verkruysse et al. [87] from the University of California at Irvine and the Norwegian University of Science and Technology at Trondheim were among the first to demonstrate that simple low-cost digital video cameras and ambient daylight illumination might be sufficient to capture a remote reflectance photoplethysmography (rPPG) waveform in controlled scenarios. The authors reported that they were able to extract both heart rate and respiratory rate.

As mentioned in Wang’s Ph.D. thesis [88], Philips Research started camera-based work on what they call the *Vital Signs Camera* as early as 2004 [88]. Starting in 2012, Philips Research has been offering this software as an app for download to a smartphone for several years. While this applet was mainly targeting private customers for fun applications, Philips Intellectual Property and Standards department recently began licensing the Vital Signs Camera for commercial customers. Intended applications include wellness products and automotive applications (see https://www.ip.philips.com/licensing/program/115).

Significant work on camera-based monitoring has been performed by Tarassenko’s group from the University of Oxford, U.K. (see, e.g., [89]). As an example, in 2014, Villarroel et al. from that group reported on heart rate estimation in neonates using CCD cameras and visible light [90]. Similarily, Guazzi and coworkers published their results on SpO_2_ imaging using RGB cameras in 2015 [91]. In order to estimate blood pressure from local pulse transit time signals, Daly presented the results of their clinical studies involving volunteers and dialysis patients in 2016 [80].

While not directly refering to the term “PPG” as the underlying measurement principle, in 2012, Wu and colleagues [92] have reported that, by employing Eulerian magnification, the subtle colour changes in the skin due to perfusion can nicely be visualized in relaxed subjects. Of course, the fascination of these visualizations results from the fact that the human eye can not see these sublte color changes directly. This explains why the corresponding videos (available on the author’s websites and on YouTube) have attracted signifcant attention.

Unfortunately, by now, a variety of names referring to remote PPG imaging exist in the literature (see Table 4).

While currently the terms *remote* PPG and sometimes *remote* PPG imaging/*remote* PPGI are becoming more and more popular, the term *PPG imaging* (PPGI) is actually much older and was originally coined at RWTH Aachen University in Germany (see [81,82,83]). From the authors perspective, this name is quite descriptive and clearly refers to the underlying methodology of using a camera for extracting physiological information. To end the Babylonian confusion on the name, it is suggested to have a naming debate in the scientific community. In any case, the abbreviation “rPPG” is an unlucky choice, as traditionally rPPG and tPPG refer to *reflective* and *transmissive* PPG, respectively.

All examples mentioned so far do not directly relate to monitoring in a car. Hence, the following subsection will look at literature with explicit automotive applications.

#### PPGI Vital Sign Monitoring in the Car

In 2014 and 2017, respectively, Zhang et al. from the Shenzhen Institutes of Advanced Technology and the Chinese University of Hong Kong, China [117,118] presented their results on PPGI monitoring of heart and respiratory rate by RGB image processing and independent component analysis (ICA) in the visible light frequency range. While the earlier publication focused on laboratory experiments, the later publication of this group included pilot measurements on one subject while driving in a real car.

In 2015, Kuo et al. [102] from Monash University in Melbourne, Victoria, Australia, reported on their results using imaging PPG. In their study, 10 participants completed an on-road driving task. A commercial heart rate monitor was used as a gold standard. As reported, IPPG produced a valid heart rate estimation for only 4 out of 10 participants.

In cooperation with Volvo (Volvo Car Corporation, Torslanda, Gothenburg, Sweden), in 2015 Rahman and colleagues from Mälardalen University in Sweden published a comprehensive review paper on the state-of-the-art in driver heart rate monitoring using cameras for the visible light range [119]. In the following year, this group suggested using a webcam for real-time heart rate monitoring [120]. While the paper focused on algorithms and lab experiments, the suggested applications included driver monitoring.

In 2016/2017, a similiar approach to heart rate detection in the car was presented by Wu et al. [107] from the National Chiao Tung University, Hsinchu, Taiwan. The authors also used RGB cameras and presented their results on lab experiments as well as test drives employing empirical mode decomposition (EMD) for filtering motion artifacts.

Similarly, in 2017, Blöcher et al. [97] from Karlsruhe Institute of Technology presented a camera-based online heart rate monitoring system for in-vehicle applications using such an off-the-shelf webcam as well. Their system reached a sensitivity of 93% under laboratory conditions (constant lightning and resting conditions). Their system was also tested under real-world driving conditions and showed encouraging results. However, due to a insufficient number of test persons (two), an additional evaluation on a larger test cohort is required to obtain statistically relevant results.

Employing cameras with active illumination in the NIR frequency has been proposed as well, but not too many reports exist thus far. In 2015, Gücüyener proposed to integrate a near infrared (NIR) camera with active LED illumination into the rear mirror and employ it for heart beat detection during driving [121]. In 2016, Tayibnapis and colleagues from the Daegu Gyeongbuk Institute of Science and Technology Daegu, South Korea, reported on using non-contact PPG in the NIR range for driver fatigue classification using support vector machine (SVM) algorithms.

### 5.3. Far Infrared Imaging (Thermography)

In contrast to VIS and NIR imaging, far infrared (FIR) imaging, also referred to as *infrared thermography* (IRT), is a passive method relying solely on the radiation emitted from the driver/passenger as a function of temperature. Hence, its biggest advantages are that no energy is transferred to the subject under test and that it can be used in complete darkness, which made this method a key technology for early adoption for military and border control applications.

While not targeting driver or passenger state monitoring at the time, early research applications of face thermography included quantifying stress and emotion [122,123] as well as vital sign monitoring (respiration) [124]. As originally demonstrated in 2007 by Pavlidis and coworkers [125], using an IRT camera to track the nostrils with a proper angle of attack allows for determining breathing rate and depth in an adult (see also [126]).

**Figure 11:**
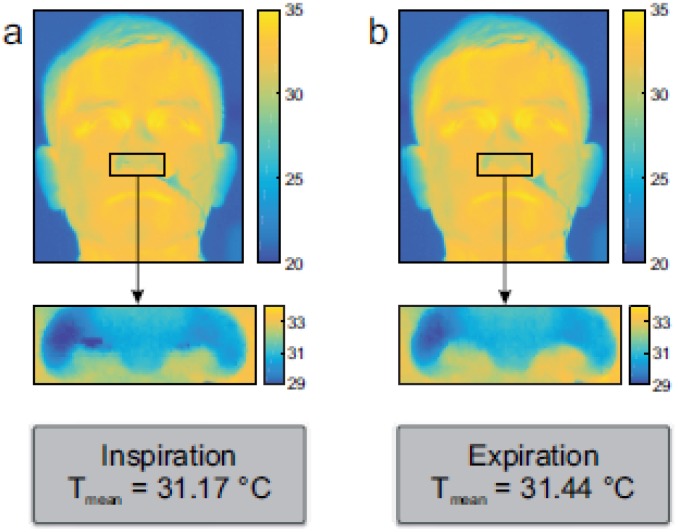
Temperature variation around the nostrils during inspiration and expiration. Thermograms of the nose during (**a**) inhalation and (**b**) exhalation from [127] (Figure 1).

However, depending on the size of the subject, the angle of attack and the individual tidal volume, the temperature changes during inspiration and expiration may be small, usually ± 1 deg. C or less, as demonstrated in Figure 11.

Note that, in this example, the mean temperatures of the region of measurement (ROM) reached 31.17 deg. C and 31.44 deg. C during inspiration and expiration, respectively. Hence, any FIR camera used for this application must be sufficiently sensitive. Recently, the RWTH Aachen group has been able to demonstrate that it is possible to follow the nostrils during movements as well [127].

While in general vital sign monitoring using IRT is currently advancing, according to our knowledge, automotive applications of IRT-based vital sign measurements have not yet been reported. However, there is at least one publication aiming at quantifying facial expressions using IRT during driving (see Section 5.4.3).

### 5.4. Other Camera-Based Automotive Monitoring

#### 5.4.1. Automotive Monitoring Using Visible Light (VIS)

With respect to early camera applications in the automotive arena, driver drowsiness detection as well as driver attention monitoring were among the first topics of interest.

As early as 2003, Smith et al. introduced an algorithm to monitor driver face orientation based on a single camera [128]. Shortly after, in 2004, Rongben and colleagues [129] reported on mouth tracking and shape analysis using CCD cameras to quantify drowsiness employing neural network (Perceptron-type) classifiers.

In 2007, Trivedi and coworkers investigated camera-based systems aiming at correlating the visual contextual information of vehicle interior and vehicle exterior [130]. Topics like occupant position and posture, driver distraction and estimation of face orientation were examined using a multi-camera system during daylight to look in and look out of the vehicle (LiLo concept). Shortly after, in 2008, Bergasa et al. reported on driver inattention analysis using a dual-camera system in the visible light range [131]. Their face detector was based on the known Viola and Jones face detection algorithm [132].

Capturing and quantifying emotions from facial images is a task that has applications far beyond the automotive scenario, but is of course quite important to access driver state. In fact, stress and emotions might be quantifiable based on vital signs (like heart and breathing rate and derivatives), but also by image processing. Correspondingly, in 2016, Manoharan and colleagues published an article on detecting stress and pain of drivers by analyzing facial expressions based on tracking dedicated fiducial points [133]. For classification of emotions, the authors employed support vector machines.

#### 5.4.2. Automotive Monitoring Using Near Infrared Light (NIR)

Near infrared camera investigations aiming at driver drowsiness detection during darkness date back at least 20 years. As early as 1994, Ueno and colleagues from the Nissan Research Center reported on their experiences in face analysis and eye tracking using a CCD camera and NIR illumination [10].

For truck driving, Grace et al. [134] reported on using the well-known PERCLOS (percentage eye closure) index, a measure of drowsiness associated with slow eye closure, in 1998. Covering the state-of-the-art a decade ago, a survey on drowsiness detection and driver state monitoring using NIR cameras was published by Wang et al. in 2006 [135]. Interestingly, these authors state that the best techniques for fatigue measurement are *“based on physiological phenomena like brain waves, heart rate, pulse rate and respiration. These techniques are intrusive, since they need to attach some electrodes on the drivers, causing annoyance to them*” [135]. Hence, at the time, NIR-based computer vision was proposed as an (inferior?) alternative instead. That way, using a CCD camera with NIR illumination as a physiological sensor fuses the advantages of both approach.

For many years, the group of Jean-Philippe Thiran at EPFL in Lausanne, Switzerland has worked on signal processing on facial images in the car. A special focus has been the quantification of emotion, see, e.g., https://transport.epfl.ch/en/research-overview/vehicles-infrastructures/intelligent-vehicles/face_analysis/emotion-detector/. Note that, in 2014, Gao et al. published on using near-infrared (NIR) cameras on the dashboard of a car for that purpose [136]. Based on the tracking of 49 face landmarks, the authors report a correct classification rate of up to 90% in their experimental setting.

In 2016, Tayibnapis and coworkers [115] presented their results on the fatigue analysis of eyes, mouth, and head-pose using a NIR camera. For face analysis, the Viola–Jones algorithm [132] was employed. In addition, heart rate and heart rate variability were extracted using no-contact PPG imaging. Hence, their proposed system features two major contributions, namely “*(1) integrating various facial feature methods using an NIR camera, and (2) measuring the driver’s physiological state with a non-contactable method*” [115].

#### 5.4.3. Automotive Monitoring Using Far Infrared Light (FIR)

As already mentioned in Section 5.3, both facial expression as well as vital sign monitoring (mainly respiratory activity) attracted the interest of several researchers quite early. Starting around the millenium, there has been continuing research activity in the area of quantifying emotions using far infrared imaging [123,137,138,139,140,141,142,143,144]. Among others, one rationale has been the observation that the perfusion of the forehead supplied by branches of the *A. facialis* changes during some emotional arousal affecting the thermal signatures of the face.

One of the first publications on using FIR imaging in the car was published by the University of Iowa in 2009 [145]. In fact, Reyes and coworkers used data obtained from *n* = 16 middle-aged test drivers during 2 h experimental drives in a driving simulator to quantify stress and emotions. Here again, perfusion changes in the eye corners and the root of the nose are key in the analysis.

### 5.5. Image Fusion

In automotive applications, the concept of image and sensor fusion dates back more than 20 years. In the beginning, imaging systems were often fused with other modalities (like radar) and were targeting mainly object recognition outside the car.

For example, in 1997, Zomotor et al. [146] described the fusion of infrared, laser and radar-based range information using a Kalman filter to improve the range detection at longer look-ahead distances required for “autonomous intelligent cruise control” (AICC) applications. A few years later, Fang et al. [147] used the fusion of radar and binocular stereo videos for a depth fusion algorithm. The improved depth information was then employed in a target segmention algorithm (like identification of other cars, pedestrians, etc.), which is required for autonomous driving. Furthermore, they demonstrated that the fusion of two camera image streams can be used for range detection, making an additional radar system obsolete.

She et al. [148] presented a system that fuses color and shape information for improved vehicle detection rates in 2004. In 2007, an interesting approach using a multi-wavelength and multi-camera approach for pedestrian detection was described by Krotosky et al. [149]. Their system employs two color and two infrared cameras in the front of the vehicle. Note that pedestrians are producing very different features in the two modalities, allowing for obtaining a unique fingerprint for pedestrian classification.

Apart from these systems directed outwards of the vehicle, systems monitoring the vehicle occupants have also been presented.

In 1999 and 2000, Pavlidis et al. [150,151] introduced a system using near-infrared imaging for vehicle occupant counting. Two near-infrared camera streams, below and above 1400 nm, were fused to segment vehicle occupants. The system was able to successfully distinguish between humans and mannequins. Possible applications include the surveillance of freeway lanes reserved for car pools.

Camera systems directed inside the vehicle can also be used to improve the safety of occupants. In 2002, Owechko et al. [152] described a vision system aiming at improving the safety of airbag deployment. Ignited airbags inflate very rapidly; they can cause severe head trauma if the vehicle occupant is too close to the device. In addition, airbags can actually kill small children in infant seats if they are placed in the front passenger seat. In such situations, the airbags must not be deployed. Owechko’s system uses two cameras and extracts four features from the images (range, edges, motion, shapes). Classifiers are used to distinguish the vehicle occupant, e.g., driver in normal position or infant in infant seat. A fusion algorithm weights the classifier outputs according to the confidence and decides about the airbag deployment.

Two years later, Trivedi et al. [153] extended the work of Owechko et al. by combining stereo and thermal video to determine the vehicle occupant position. As discussed before, thermal imaging has the benefit that it does not require illumination.

In 2004, Gyaourova et al. [154] combined visible and infrared images to improve face detection in people wearing eyeglasses. Infrared imaging has the benefit of being independent of illumination, but cannot pass through spectacles. On the other hand, visible imaging operates through eyeglass, but requires illumination. Therefore, a combination of both modalities can improve coverage rates. This, of course, is important to determine the PERCLOS ratio (see above) or the direction of view.

A yawning detection as a sign of driver fatigue was presented by Li [155] in 2009. It uses a real-time adaptation of the region of interest (ROI) of a camera. A low-resolution camera captures a larger region of the driving compartment. A face detection is performed on that image stream and a second camera is directed onto the mouth of the driver according to the detected face. It is argued that, by this approach, the driver’s mouth can be recorded at a higher resolution, providing a more accurate measure of fatigue.

Capturing and quantifying emotions from facial images is a task that has applications far beyond the automotive scenario, but is of course quite important to access driver state. For generic facial expression recognition, in 2016, Corneanu and coworkers [156] published a comprehensive survey on available databases containing faces in the RGB, NIR and far infrared range. Interestingly, there is far more data publically available in the RGB band than for IRT. A few databases (like NVIE and KTFE) offer labeled images in both frequency bands [156]. Recently, Wang et al. presented a deep learning approach for fusing visible and infrared images for facial expression recognition [157].

### 5.6. Video Motion

The general principle of ballistocardiography (BCG) has already been introduced in Section 4 of this review paper. While some groups including Verkruysse et al. [87] and Blanik et al. [158] have reported on BCG artifacts during PPG imaging before, Moco and colleagues [95] were the first to systematically quantify BCG artifacts for PPGI of the face.

However, treating the BCG component as an artifact in PPGI is not the only option in making use of this information. When heart rate estimation and not local PPG monitoring is of interest, the BCG artifact in image streams may actually be utilized as a separate signal source.

With respect to using cameras as a BCG sensor, in 2013, Balakrishnan and colleagues from the M.I.T. demonstrated that BCG can also be performed by video stream analysis [159]. In fact, these computer scientists demonstrated that proper face detection (OpenCV 2.4 Viola Jones algorithm [160]), subsequent OpenCV Lucas Kanade tracking of feature points and PCA decomposition allows for finding and magnifying the normally unnoticeable motions of the head in temporal image sequences, in that way similar to the color magnification performed by Wu et al. [92].

In 2015, Hoog Antink and colleagues have shown that robustness and coverage of heart rate estimation can be increased by fusing PPG and BCG information extracted from visible video streams [161].

Despite motion artifacts that can be expected during driving, an automotive application of camera-based BCG seems possible, but—according to our knowledge—has not been demonstrated thus far.

## 6. Magnetic Induction

In general, human tissue is an inhomogeneous and anisotropic electrical medium. Within the thorax of a human, the distribution of electrical impedance is modulated by cardiorespiratory activity due to organ motion and deformation as well as fluid displacement. Magnetic Induction (MI) monitoring is a no-contact method to measure this intrathoracic impedance distribution.

**Figure 12:**
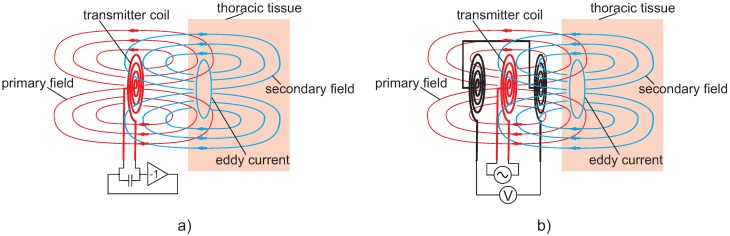
Principle of magnetic induction monitoring. (**a**) single coil approach with frequency modulation and (**b**) multi-coil approach based on a gradiometer.

Basically, the principle is based on electromagnetic coupling between a coil and the thorax in its vicinity as illustrated in Figure 12.

When an alternating current (typically 10–20 MHz) flows through the air coils, this current induces a primary field affecting the tissue underneath, which results in eddy currents flowing on closed pathways perpendicular to the field. The eddy currents are modulated in amplitude, direction and orientation by air and blood volume changes, displacement of organ boundaries, and microscopic processes. As with any current flow, the eddy currents create a secondary field, which carries the information of the modulation. The penetration depth of this active method is estimated to be approximately the diameter of the coil. A brief introduction to the physics of the signal generation can be found in [162].

Single coil systems typically operate as part of a resonant oscillator circuit. The coil serves as a transmitting as well as a receiving coil and is a frequency-determining part of the oscillatory circuitry. When the coil’s reflective impedance changes due to the secondary field affecting the primary one, this will cause a change in the oscillatory frequency. This frequency change then correlates with physiological activity and is utilized as the sensor output signal.

By contrast, multi-coil approaches feature several coils, typically one transmitter and one or two receiver coils. In multi-coil arrangements, it is necessary to position the receiver coils such that the primary field induction is as small as possible while the secondary field induction should be as large as possible. When only one receiver coil is used, it has to be brought into an orthogonal orientation with the transmitter coil in order to ensure that the primary field does not induce any voltages into the receiver coil. When two receiver coils are used, they are typically connected as gradiometers such that the primary field is (almost) completely compensated. In [163], planar gradiometer arrangements have been proposed, but they have not been integrated into vehicles yet.

**Figure 13:**
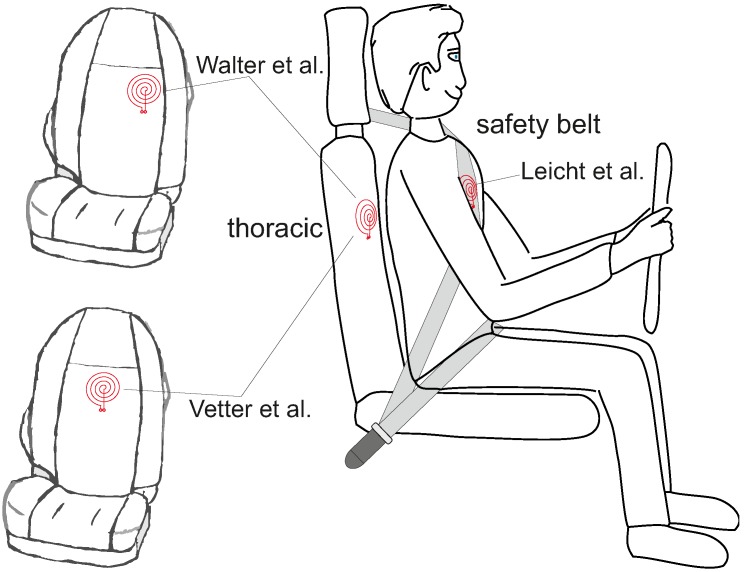
Coil locations of published in-car MI monitoring systems.

To date, three articles have been published which report on the use of MI monitoring within a car. Figure 13 gives an overview about the different locations where these MI sensors were located.

**Figure 14:**
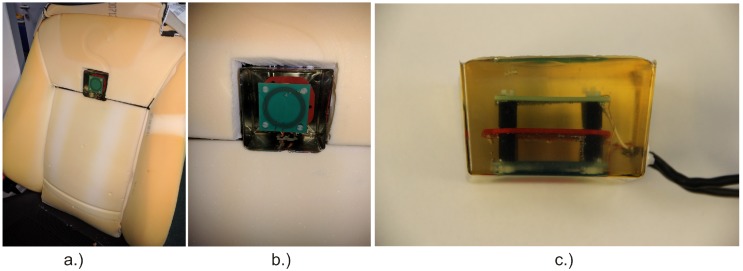
Gradiometer sensor inside a resin block integrated into the driver seat (**a**), modified and extended from [165]. Middle and right figure are magnified views of the sensor from top (**b**) and from the side (**c**) showing the spatial structure of the gradiometer.

In 2011, Walter et al. presented the integration of a single coil MI sensor into the backrest of a vehicle seat [74] and demonstrated its usability for respiration monitoring. While these results were preliminary, this was the first time that the MI technique has been employed inside a vehicle. In 2017, Leicht et al. presented the *PhysioBelt* device [164], which comprised an MI device with a flexible coil sewn into the seat belt. In the same year, Vetter et al. [165] reported on progress with gradiometer technology for automotive integration. This new design features a gradiometer approach with one sending and two receiving coils forming a vertical gradiometer (see Figure 14).

While the former two devices only measured respiration, the latter MI device was also able to record a cardiac signal. While these results are preliminary and have not yet been thoroughly tested in larger studies, they at least demonstrate both ventilation and cardiac contraction can be observed in a MI signal measured in the backrest, with ventilation dominating by one order of magnitude in signal strength.

## 7. Radar-Based Methods

The term *radar* actually is an abbreviation refering to *radio detecting and ranging*. This technique uses high-frequent electromagnetic waves that are emitted from a transmitter (Tx) and reflected by the chest surface thus carrying information on human chest displacement and, to some extent and as a function of penetration depths, on inner organ movements. The reflected waves are sensed by a receiver (Rx) and analysed, e.g., in shift of phase, etc. Radar methods are mainly carrying information on movement, which can of course be caused by both respiratory and cardiac activity. As mentioned in [166], the heart can typically cause chest displacements up to ± 0.1 mm in relaxed humans (see also [167]), and respiration displacements of between 0.1 mm and several millimeters. Due to this, radar methods provide similiar information as ballistocardiography (BCG) methods.

There are two general radar techniques: the pulsed *ultra wide-band* (UWB) radar, often with a separate transmitter and receiver antenna, which is based on measurements of time-of-flight and derived quantities, and the *continuous wave* (CW) radar which basically relies on the Doppler effect.

In the U.S., the frequency range for UWB radar is regulated by the Federal Communications Commission (FCC) spans 3.1 GHz to 10.6 GHz. By contrast, in Europe, the allowed frequencies range from 3.4 GHz to 4.8 GHz and from 6 GHz to 8.5 GHz [168].

Vital sign monitoring in the frequency range of a few GHz is possible and has been demonstrated for UWB radar mode in many applications [169], including rescue missions (to *locate human subjects burried under earthquake rubble* or *being covered under snow* after avalanche accidents [170]). However, in this frequency range, antennas have been quite bulky and also the allowed transmission power is rather limited. Higher frequencies (like microwave radar in the near 60 GHz ISM band) offer some advantages such as higher sensitivity and smaller antennas [171]. However, at such high frequencies, the penetration depth of electromagnetic radiation into biological tissue is almost negligible and the radar system is mainly a BCG sensor. While this may be acceptable for respiration tracking, heart-related motions are very small and hard to detect.

Early attempts to use radar for measuring vital signs date back to the 1970s. In fact, in 1971, Caro and Bloice presented a 10 GHz radar system mounted on top of an infant incubator aiming at apnea detection [172].

In 2009, Ichapurapu and colleagues reported an accuracy of ± 1 heartbeat per minute for heart rate detection using a 2.4 GHz CW radar system [173]. Similiarly, in 2009, Massagram and colleagues demonstrated in 12 healthy subjects that heart-rate variability (HRV) and respiratory sinus arryhtmia (RSA) can be monitored using continuous Doppler radar in the 2.4 GHz range [174].

In 2010, Lazaro and colleagues shared their experiences using an impulse radio UWB radar system prototype manufactured by Geozondas, Lithuania, for vital sign monitoring [166]. In this work, the authors emphasized at the mathematics used for heart and respiration signal extraction. They also reported that the measurements were in compliance with the FCC mask (allowed dissipation power) almost everywhere in the 3.1 to 10.6 GHz frequency range.

In 2011, Scalise et al. presented a measurement method for respiration using a 6 GHz CW-radar system [175]. Despite successfully detecting respiration activity up to 2.5 m, the use in automotive environments might be difficult since the system uses a large double ridge horn antenna.

Bechet et al. [176] reported in 2013 on their results improving the signal quality of CW Doppler radar vital sign tracking at 2.4 GHz using the MUSIC (Multiple Signal Classification) algorithm.

**Figure 15:**
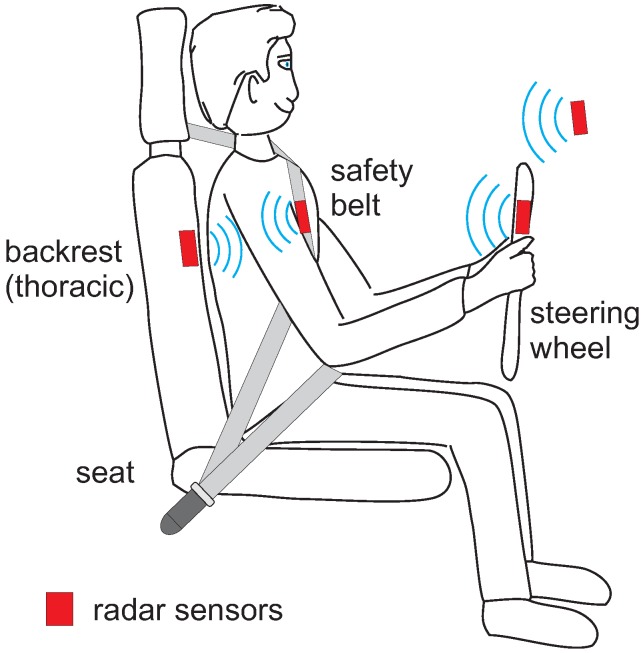
Potential locations of radar sensors inside a car.

For automotive applications, various sensor locations are possible, including integration into the cockpit, the safety belt, the steering wheel and into the backrest (see Figure 15).

In 2015, Vinci et al. proposed using continuous wave (CW) radar sensors for monitoring vital signs while driving [177]. The authors of this paper investigated two measurement scenarios: in the first scenario, a 24 GHz six-port configuration of the radar system is placed approximately half a meter in front of the driver, with the antenna pointing directly on its thorax. This distance between sensor and driver would allow future integration of the antenna, for example into the steering wheel. In the second measurement scenario, the same radar sensor has been installed on the backrest of the car seat (which is more difficult for such high frequencies, as mentioned above). Here, the authors intend to create a “biometric driver seat” while at the same time eliminating the necessity of a sensor integrated in the steering wheel. Therefore, the radar antenna is not aiming on the thorax of the person under test, but on his or her back.

A similiar approach was proposed by Lee and colleagues in 2016 [178]. Similiar to Vinci’s second scenario, the authors proposed to place a 24 GHz CW sensor in the backrest of the car seat. These authors also point out that, using CW radar, motion artefacts may significantly interfere with the frequency characteristics of the displacement signal and may hence be difficult to extract by signal analysis in the frequency domain (FFT).

In 2016, Izumi et al. presented a 24 GHz microwave Doppler sensor circuit placed on the chest of a volunteer sitting in a car seat. As demonstrated, the location is proper for seat belt integration [179]. Very recently, Schires and colleagues [180] demonstrated feasibility of heart and respiratory rate tracking using a new UWB radar design placed in the backrest of a car seat. A *Ventricorder Impulse Radar* module manufactured by the Norwegian company Novelda (Novelda AS, Gjerdrums vei 8, 0484 Oslo, Norwegen) was employed. In order to increase the penetration depth of the electromagnetic impulses, the authors chose a carrier frequency of 3.9 GHz using pulses of approximatively 2 GHz bandwidth.

## 8. Challenges of In-Vehicle Measurements

### 8.1. Motion Artifacts

When measuring biosignals unobtrusively, coverage and accuracy can be relatively low compared to clinical modalities. By using methods for sensor fusion, both quantities can be improved by exploiting redundancies in multiple measurement channels. When considering contact-free monitoring of vital signals, motion artifacts always pose a problem [181]. Compared to classical, cable-bound sensing, they are always more severe.

In particular, radar-based measurement systems are susceptible to motion artifacts, since they derive a vital sign by means of a distance measurement. To cope with this problem, Tang et al. developed a radar system using a transmit and re-transmit approach. The system uses two antennas, one being located in front and one being located behind the patient [182]. Vital signs, in particular respiration and heart rate, will move the patients towards or away from both antennas at the same time (in phase), while artifact movements will cause a out-of-phase movements (towards one antenna and away from the other antenna, and vice versa). The system makes use of these circumstances by first transmitting and receiving a signal with one antenna and then feeding the received signal into the other antenna. When received by this antenna, the signal contains in-phase signal parts associated with the vital signs (constructive interference) and out-of-phase signal parts from the artifact movement (destructive interference). Thus, despite artifact movement, the system can successfully detect heart and respiration activity.

Furthermore, most other modalities also have specific problems. Camera-based methods, for example, only work if the subject under test is in the field of view. Capacitive biopotentials may suffer from common mode problems resulting from maladjusted clothing combinations (e.g., polyester–cotton, silk–cotton, etc.).

Ottenbacher and Heuer [183] have analysed the contribution of lateral and transversal changes in distance between electrode and skin. While global triboelectricity equally affects all electrodes and acts as a common-mode interference, local transversal changes in distance directly affect the coupling capacity of the electrode–skin combination. This can be modelled as a time-dependent coupling capacity.

Wartzek et al. demonstrated in 2011 that additional factors contributing to motion artifacts in capacitive ECG are local triboelectric effects on the electrode–body interface. These seem to depend on the relative distance in the triboelectric series [46]. If combined with lateral movements, this relative distance determines how sensitive different fabric combinations are to common mode signals due to local triboelectricity.

Several groups have proposed algorithmic and technical methods to fight motion artifacts. In 2011, Eilebrecht and coworkers proposed the use of adaptive filters for artifact cancellation [184]. Afterwards, Eilebrecht et al. [185] and later Serteyn and coworkers [181] proposed to analyse the electrode–skin interface by injecting high frequent test currents. This means that the interface is characterized by some sort of impedance measurement. In 2016, Lee et al. demonstrated that motion artifacts in radar systems can be greatly reduced using multiple signal classification (MUSIC) [178]. The authors investigated three different driving conditions: halted vehicle, driving at 60 kmh and driving at 80 kmh. Using a classical, FFT-based approach, the average error rate regarding the heart rate was below 3 bpm when the vehicle was halted, but excessive (approx. 25 bmp) at 60 and 80 kmh. Using the MUSIC approach, the error rate was reduced in all three driving conditions and stayed below 3 bpm.

### 8.2. Variable Light Conditions

Camera-based vital sign monitoring requires, with the expection of cameras operating in the far infrared spectrum, light that can be detected by the camera. For in-vehicle applications, sufficient light cannot be guaranteed, for example while driving at night. Artificial illumination is therefore required, which can blind the driver in case light in the visible range is used and is thus a traffic risk.

A solution may be the use of near infrared light which cannot be detected by the human eye, but can be detected by camera systems. However, it is not yet decided whether NIR light is harmful to the human eye. NIR light can damage both the cornea by thermal injury and the lens by cataractogenesis. Devereux et al. [186] argue that, under daylight conditions, NIR light is always accompanied by visible light. The eye will regulate light intake by closing the iris and protect itself. However, under night conditions, the iris will be widely open, allowing artifical NIR illumination to enter the eye unattenutated. Damage to the eye after long-term exposion is therefore conceivable.

Apart from the absence of light and hence the requirement for artifical illumination, varying light conditions can also pose a challenge, e.g., driving through an avenue with large trees or shadows from other cars can rapidly change the signal recorded by the camera. Hence, a correction algorithm is required. For example, Jeanne et al. [187] applied spatio-temporal filtering and achieved a correlation with the reference of 0.99. Lee et al. [188] used knowledge about the external light source to correct the camera video.

## 9. Sensor Fusion

As described in Section 8, the measurement of physiological signals in a car can be a difficult challenge due to motion artifacts not only induced by the vehicle but also the driver himself. This is even more pronounced, when using unobtrusive sensor techniques. By applying sensor fusion, some parts of this challenge might be mitigated. The term sensor fusion here stands for the use of multiple sensors at different locations or the use of different sensor modalities at the same time or a combination of both.

Applying sensor fusion would allow

- artifact compensation, e.g., by utilizing adaptive filtering with one sensor serving as the noise signal.
- source separation, for example by applying algorithms based on statistical dependencies between the signals (like independent component analysis).
- coverage rate enhancement, e.g., by utilizing multiple sensors measuring the same vital sign and, therefore, increasing the probability that this vital sign is obtainable at a point in time.

As mentioned before, in 2015, Hoog Antink and colleages from RWTH Aachen University have demonstrated that fusing unobtrusive measurements indeed improves coverage and robustness of vital sign estimation [161]. In their experimental setup, the authors actually fused camera-based (web cam for BCG and PPG) information and one-dimensional signal information (BCG in a seat).

Two years later, these authors further demonstrated [189] that the fusion of multiple signals (PPG, BCG, cECG, MI) can further improve vital sign estimation of seated subjects, in particular when motion artifacts are present.

Since the assessment and evaluation of several driver states like stress level, sleepiness or emotions require a variety of different physiological indicators, sensor fusion seems mandatory for driver state estimation. Several studies about driver state estimation have been conducted, using measurement setups with conventional, i.e., obtrusive, mainly electrode-based, sensors [7].

**Figure 16:**
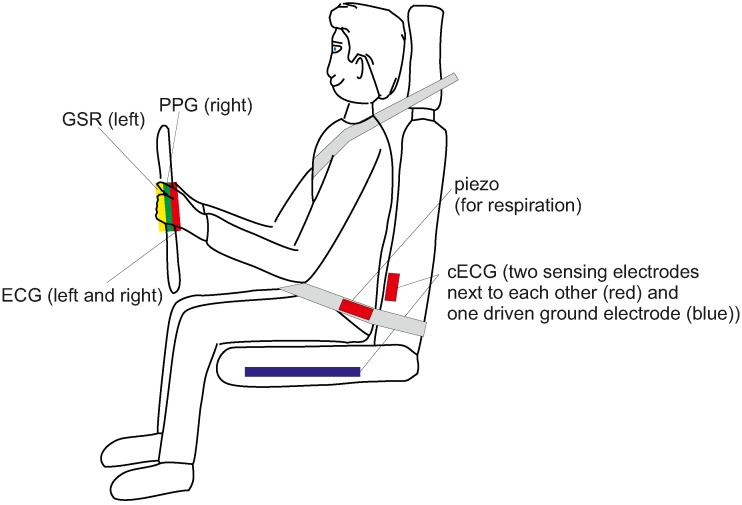
Early concept for sensor fusion inside the *U-car* [34].

Already in 2009, Baek et al. from Seoul National University and some other research organisations in Korea proposed to combine several unobtrusive sensing modalities in the car [34]. In particular, the authors presented a demonstrator (a test vehicle called the *U-car*) in which they simultaneously measured cECG in the backrest of the seat, ECG, PPG and galvanic skin response (GSR) in the steering wheel and respiration using a piezoelectric sensor in the seat belt. Their concept is shown in Figure 16.

In a small study involving *n* = 4 young healthy volunteers, driver stress levels were assessed during night driving, cold environment, hot environment, noisy environment, narrow alleys and with simultaneous mental loads (arithmetic operations). For quantification, both heart rate variability (HRV) indices as well as pulse arrival time (PAT) were measured.

A similar system was presented by Vavrinsky et al. in 2010 [30] and 2012 [31]. The system comprises two electrodes for ECG measurement, two GSR electrodes and two newly designed IDAT microsensors. All devices are built into the steering wheel. The IDAT sensors integrate an inter-digital array (IDA) [190] and a temperature sensor (T) into one small device and can be used for the measurement of skin temperature, GSR and heart pulse. The system also includes a pressure sensor integrated into the seat measuring respiration, heart pulse and driver weight. In addition, a camera-based emotion recognition system was integrated, but no results were presented. While this system incorporates several sensors for the same modality, sensor fusion still needs to be demonstrated.

## 10. Conclusions

In this review, special attention was given to unobtrusive and no-contact sensors for vital sign monitoring that can be placed inside the car, i.e., in the seat, the safety belt, the steering wheel or the cockpit. Based on different physical principles, many technologies exist and were introduced. Table 5 compares different technical aspects of these sensors including the question of active energy injection, costs, allowable distance between subject and device.

According to the number of car-related publications per technology, the former and present efforts to incorporate these technologies into the car is not equally distributed among them. While the seat-integrated techniques ECG, cECG and BCG have been applied in the automotive environment quite extensively, camera-based techniques, although already at a high readiness level, have not been investigated to the same extent. This might be due to their comparably high costs and the potential conflict with drivers feeling observed and violated in their privacy.

In this review, pilot applications and demonstrators as well as commercial products (where existing) were presented and discussed. However, measurement scenarios using any of the upcoming wearables, alone or in combination with fixed sensors, were not covered. In the long run, sensor fusion with such sensors is considered beneficial and should be further investigated. Today, available no-contact sensors share the problem of motion artefacts. For some of the technologies, additional challenges exist (like triboelectricity for cECG and varying light conditions in the visible and NIR frequency range). To increase robustness for such delicate measurement scenarios, sensor/signal fusion is one of the options, next to motion artifact cancellation. This requires multi-sensor and multi-channel measurement setups with redundant as well as complementary sensor arrangements.

## Figures and Tables

**Table 1:** Timeline of the development of low-contact ECG systems.

1967	First description of insulated electrodes (Richardson, [35])
1993	cECG in objects of daily life (Ishijima, [36])
2004	cECG in a bathtub and toilet seat (Lim, [37], Kim [38])
2006	Integration of cECG into a chair (Lim, [39])
2007	cECG integrated into beds (Lim, [40])
2008	First integration of cECG into an automotive seat (Leonhardt, [42])
2008	Textile cECG electrodes in an automotive seat (Chamadiya, [43])
2011	Evaluation of cECG during test drives (Eilebrecht, [44])
2011	Measurement performance and coverage rate tested during highway and city traffic (Wartzek, [45])
2011	Triboelectricity and motion artifacts identified as factor for robustness (Wartzek, [46])
2011	cECG evaluated as a technology for passenger monitoring in airplanes (Schumm, [47])
2012	Introduction of a quality label to describe cECG signal quality (Schumm, [47])
2012	Textile seat cover for retrofitting seats with cECG systems (Schneider, [48])
2012	cECG used for heart rate variability analysis (HRV) (Jung, [49])
2012	First cECG sensor system commercially available: EPIC system by Plessey Inc.
2015	Active humidification further evaluated (Fong [50], Weder [51])
2016	WARDEN seat cover based system for retrofitting (Plessey, [52])
2017	Closed-loop humidification for artifact suppression (Leicht, [53])
2017	IMEC sensor system fusing cECG and radar (IMEC, [54])
2018	Clinical trial to evalute cECG as a diagnostic tool for heart attack survivors (Leicht, [55])

**Table 2:** Comparison of different cECG systems integrated into car seats.

System	Year	References	Electrode Properties			Special Properties
			#	Shape	Ground	
SMART Test Vehicle	2008	[42]	2	rectangular	in backrest	isolated (coated) metal electrodes
Daimler S-class seat	2008	[43]	2	round	in seat plane	
Ford S-Max seat	2011	[44]	6	rectangular	in seat plane	multi-electrode system
Daimler Test Vehicle	2011	[58]	2	rectangular		textile electrodes
Audi Q5	2012	[48]	2	rectangular	in backrest	textile electrodes, removable seat cover
Car seat	2012	[49]	2	rectangular	in seat plane	
Ford S-Max seat	2014/2017	[53,60]	2	rectangular	in seat plane	textile electrodes, release of humidity
Plessey EPIC^TM^ seat	2014	[59]	3	round		
Ford S-Max Test Vehicle	2015	[61]	6	round	in seat plane	deep-drawn electrodes
Plessey WARDEN^TM^	2016	[52]	6	rectangular	backrest, built-into main unit	removable seat cover
IMEC car seat	2017	[54,65]	6	round		up to 64 electrodes possible

**Table 3:** Comparison of different camera-based monitoring systems.

Name	Freq. Range	Abb.	Price	Energy	Special Properties
Visible Light (Vis)	350 nm–740 nm	VIS	cheap	ambient light (passive) or	ambient light may fluctuate,
				active illumination	passive mode does not work at night
Near Infrared (NIR)	740 nm–1 μm	NIR	cheap	active illumination	works at night, long exposure may exhaust the retina
Far Infrared (FIR)	3 μm–5 μm	MWIR	expensive	passive	works at night, but glasses are not transparent
Far Infrared (FIR)	8 μm–14 μm	LWIR	expensive	passive	works at night, but glasses are not transparent

**Table 4:** Comparison of different names for PPG imaging.

Name	Abbreviation	First Mentioned	References (First Author, Year)
PPG imaging	PPGi/PPGI	2000	Blazek 2000 [81], Wu 2000 [82], Hulsbusch 2002 [83], Blazek 2006 [93],
			Karlen 2015 [94], Moco 2016 [95], Blazek 2017 [96], Blöcher 2017 [97]
Vital Signs Camera	VSC	2004	Philips Research 2004 [98]
imaging PPG	iPPG/IPPG	2007	Zheng 2007 [86], Hu 2008 [99], Sun 2014 [100], Kamshilin 2015 [101],
			Karlen 2015 [94], Kuo 2015 [102], Blackford 2016 [103], Sun 2016 [79]
remote PPG (imaging)	n.a.	2008	Verkruysse 2008 [87], McDuff 2015 [104]
remote PPG	rPPG	2015	Kwon 2015 [105], Gastel 2015 [106], Wu 2016 [107], Gastel 2016 [108]
DistancePPG	n.a.	2015	Kumar 2015 [109]
video-based HR	n.a.	2015	Choe 2015 [110]
camera-based PPG	cbPPG	2015	Wedekind 2015 [111], Rasche 2016 [112], Kevat 2017 [113]
non-contact PPG	ncPPG	2016	Butler 2016 [114], Tayibnapis 2016 [115]
video-PPG	vPPG	2016	Iozzia 2016 [116]

**Table 5:** Comparison of different ambient unobtrusive cardiorespiratory monitoring techniques by their technical aspects. Part of the information in columns 5 to 7 have been taken and adapted from [3]. RL stands for technology *readyness level* estimated by the authors.

Sensor Technique	Type of Contact	Measured Quantity	Energy Injection	Distance	Sensitivity to Positioning	Costs	RL	In-Car Use
ECG (steering wheel)	galvanic	electric biopotential	no	0	+	o	+	[20,21,24,25,27,28,29,31,32,33]
cECG	capacitive	electric biopotential	no	mm	+	o	o	[42,43,44,48,49,52,58,59,60,61]
BCG	mechanical	displacement, force	no	0	–	o	o	[42,74,75,76,78]
Video motion	optical	displacement	no	m	–	+	+	not yet
PPG (steering wheel)	optical	photon absorption	yes	mm	o	–	+	[23,25,26]
PPGi	optical	photon absorption	yes	m	–	+	o	[115,121]
Thermography	optical	radiation, temperature	no	m	–	++	+	[124,126]
MI	electromagnetic	electric bioimpedance	yes	cm	o	o	–	[74,164,165]
Radar	electromagnetic	displacement, velocity	yes	m	–	o	o	[177,178,179,180]

## Abbreviations

The following abbreviations are used in this manuscript:

BCG	ballistocardiography
cbPPG	camera-based photoplethysmography
cECG	capacitive electrocardiogram
CW	continuous wave
ECG	electrocardiogram
EMD	empirical mode decomposition
EMFi	electromechanical film
EPIC	electric potential integrated circuit
FCC	Federal Communications Commission
FIR	far infrared light
HRV	heart rate variability
ICA	independent component analysis
iPPG	imaging photoplethysmography
IRT	infrared thermography
ISM	industrial, scientific and medical
KIT	Karlsruhe Institute of Technology
LED	light-emitting diode
LWIR	long wave infrared light
MI	magnetic impedance
MWIR	medium wave infrared light
ncPPG	non-contact photoplethysmography
NIR	near infrared light
PAT	pulse arrival time
PD	photodetector
PERCLOS	percentage eye closure
PPG	photoplethysmogram
PPGI	photoplethysmography imaging
PTT	pulse transit time
QL	quality label
RADAR	radio detecting and ranging
rPPG	reflective or remote photoplethysmography
RSA	respiratory sinus arrhythmia
Rx	receiver
SpO_2_	oxygen saturation
SVM	support vector machine
tPPG	transmissive photoplethysmography
Tx	transmitter
UWB	ultra-wide band
VIS	visible light
